# A Novel GAN-Based Synthesis Method for In-Air Handwritten Words

**DOI:** 10.3390/s20226548

**Published:** 2020-11-16

**Authors:** Xin Zhang, Yang Xue

**Affiliations:** School of Electronic and Information Engineering, South China University of Technology, Guangzhou 510641, China; 201821011494@mail.scut.edu.cn

**Keywords:** in-air handwriting recognition based on inertial sensors, adversarial learning, corpus, splicing module, generating module

## Abstract

In recent years, with the miniaturization and high energy efficiency of MEMS (micro-electro-mechanical systems), in-air handwriting technology based on inertial sensors has come to the fore. Most of the previous works have focused on character-level in-air handwriting recognition. In contrast, few works focus on word-level in-air handwriting tasks. In the field of word-level recognition, researchers have to face the problems of insufficient data and poor generalization performance of recognition methods. On one hand, the training of deep neural learning networks usually requires a particularly large dataset, but collecting data will take a lot of time and money. On the other hand, a deep recognition network trained on a small dataset can hardly recognize samples whose labels do not appear in the training set. To address these problems, we propose a two-stage synthesis method of in-air handwritten words. The proposed method includes a splicing module guided by an additional corpus and a generating module trained by adversarial learning. We carefully design the proposed network so that it can handle word sample inputs of arbitrary length and pay more attention to the details of the samples. We design multiple sets of experiments on a public dataset. The experimental results demonstrate the success of the proposed method. What is impressive is that with the help of the air-writing word synthesizer, the recognition model learns the context information (combination information of characters) of the word. In this way, it can recognize words that have never appeared in the training process. In this paper, the recognition model trained on synthetic data achieves a word-level recognition accuracy of 62.3% on the public dataset. Compared with the model trained using only the public dataset, the word-level accuracy is improved by 62%. Furthermore, the proposed method can synthesize realistic samples under the condition of limited of in-air handwritten character samples and word samples. It largely solves the problem of insufficient data. In the future, mathematically modeling the strokes between characters in words may help us find a better way to splice character samples. In addition, we will apply our method to various datasets and improve the splicing module and generating module for different tasks.

## 1. Introduction

Human–computer interaction (HCI) [1,2,3] is a new research hotspot. In the past, HCI methods were mostly limited to hardware devices, such as touch screens, keyboards, etc. With the continuous development of HCI systems, many promising solutions have emerged. The in-air handwriting system based on inertial sensors introduced in this work is one of them. In-air handwriting means writing characters or words freely in 3D space, which is a smarter way of HCI. In recent years, with the development of MEMS (micro-electro-mechanical systems), smartphones and wearable devices with built-in inertial sensors have become more and more popular. Therefore, in-air handwriting systems based on inertial sensors have attracted wide attention from many researchers.

In the field of in-air handwriting, most of the previous works focused on character-level in-air handwriting recognition (char.-IAHR) [4,5]. Few studies involve word-level in-air handwriting recognition (word-IAHR). There is a big difference between char-IAHR and word-IAHR. Char-IAHR is a classification task based on a single character. Usually, this is a relatively simple task and does not require much training data. Using thousands of samples, the char-IAHR model can usually achieve good generalization performance [6,7,8]. Unlike char-IAHR, word-IAHR not only needs to consider the characteristics of the current character, but also the influence of the characters before and after it. In other words, the word-IAHR model not only learns to classify characters, but also learns the combinations of characters. It is a more complicated task and usually requires a large amount of training data. In practice, for in-air handwriting system, outputting words has broader application prospects than outputting single characters. Therefore, the research on word-level in-air handwriting is more meaningful.

However, the word-IAHR task faces two difficulties. Firstly, the training of deep neural networks usually requires a particularly large data set. Adequate data collection is a solution, but it will take a lot of time and money. Furthermore, it is difficult to collect a well-distributed dataset. Secondly, a deep recognition network trained on a small data set can hardly recognize samples whose labels do not appear in the training set. In the word-IAHR task, the classification of the current character depends not only on its own characteristics, but also on characteristics of contextual characters. The recognition model is likely to fail to give the correct answer if the combination of characters is fresh to the model. Therefore, it is really important to introduce the corpus in the training process. For example, we use the dataset “economics” to represent a dataset whose vocabulary belongs to the field of economics. Generally, if training is performed on the dataset “medicine”, the recognition model will perform poorly on the dataset “economics”. This is because there are many professional words in the field of economics, and their character combinations are very rare in the field of medicine.

Therefore, it is of practical significance to apply a method to synthesize realistic word-level in-air handwritten samples. This will greatly solve the problem of insufficient data in the word-IAHR task.

**Figure 1:**
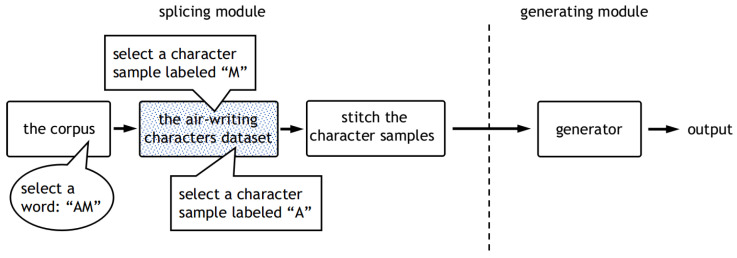
Pipeline of the air-writing word synthesizer. It can be divided into a spicing module and a generating module. The splicing module splices the character samples according to the corpus. The generating module takes in the spliced samples and generates realistic word samples.

In this work, we propose a novel synthesis method for in-air handwritten words, named the “air-writing word synthesizer”. Figure 1 shows the pipeline of the air-writing word synthesizer. When the word synthesizer is well trained, the output of the generating module will be used to train a word-IAHR network.

The air-writing word synthesizer consists of two modules, namely the splicing module and the generating module. (1) The splicing module splices the character samples according to the corpus to get the spliced samples. Note that the corpus is a collection of words, which can provide a rich combination of characters for the proposed method. The splicing module is not trainable. It consists of a series of steps. The specific splicing steps will be introduced in Section 3.2. (2) The generating module takes in the spliced samples and generates realistic word samples. We regard the conversion from the spliced samples to realistic word samples as a domain-transferring task. The generating module is actually a generator in a generative adversarial network (GAN). This is a trainable network that is trained by adversarial learning. In order to enable the network to process time series, we redesigned the network structure and loss function. The details of the generating module, including training details, network architecture, etc., will be introduced in Section 3.3.

The air-writing word synthesizer proposed in this paper mainly includes the following characteristics:

- The air-writing word synthesizer is a two-stage synthesis method. It creatively combines the splicing module and the generating module to achieve the best performance. Due to its unique design, the air-writing word synthesizer can synthesize a large number of realistic word samples based on limited character samples and word samples.
- The splicing module introduces an additional word set, namely the corpus. The splicing module synthesizes word samples by splicing character samples according to words in the corpus. In our design, the corpus provides important guidance for the combination of characters.
- To make the spliced samples more real, adversarial learning is adopted in the training process of the generating module. In order to process word samples of arbitrary length, we use a fully convolutional U-Net [9] generator and a Markovian discriminator [10]. This design also makes our model pay more attention to the details of the generated samples.
- In the training process of the generating module, the distance loss is adopted to maintain semantic consistency.

In particular, we design a word recognition model to verify the effectiveness of the air-writing word synthesizer on a public dataset. Experiments show that the recognition model trained on synthetic data performs well on samples with fresh labels. To a certain extent, the proposed method solves the problem of insufficient data of in-air handwritten words.

We propose a novel and promising GAN-based synthesis method for in-air handwritten words. We believe that this work will provide some new inspiration for other researchers.

## 2. Related Work

### 2.1. In-Air Handwriting System

As a new hotspot in the field of human–computer interaction, many kinds of in-air handwriting systems based on different sensors have emerged. Most famous air-writing systems are mainly based on two types of sensors, namely optical and depth cameras [11,12,13] and inertial sensors. The former allows users to write virtual text directly on the desktop or in the air with their fingers and uses optical and depth cameras to record hand movement information. The latter allows users to write freely in the air using a handheld device with built-in inertial sensors and uses an accelerometer and gyroscope to capture motion information. Inertial-sensor-based systems are not affected by ambient light intensity or camera coverage, so they can be applied to more scenarios.

**Figure 2:**
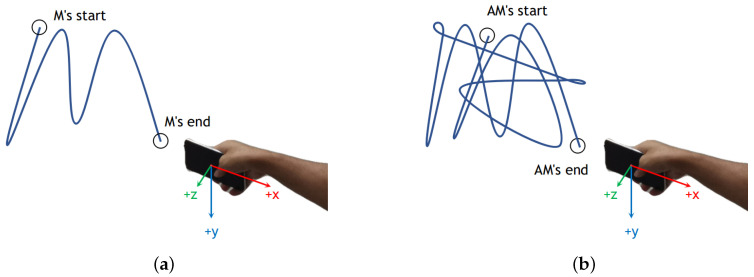
In-air handwriting system: (**a**) writing a character sample “M” in the air; (**b**) writing a word sample “AM” in the air. The red, blue, and green arrows refer to the x-axis, y-axis, and z-axis of the inertial sensor in the smartphone, respectively.

In this article, we mainly discuss an in-air handwriting system based on inertial sensors. As illustrated in Figure 2, inertial sensors are integrated into smartphones. The red, blue, and green arrows refer to the *x*-axis, *y*-axis, and *z*-axis of the inertial sensor (accelerometer and gyroscope), respectively. Figure 2a and Figure 2b respectively illustrate the process of writing the character sample and word sample in the air. Note that the writing process is completed in one stroke regardless of character sample or word sample.

### 2.2. Generative Adversarial Networks

Generative adversarial networks (GANs) [14] were proposed by Ian Goodfellow. A GAN is composed of a generative module and a discriminative module. The discriminative module learns to determine whether a sample is from the model distribution or the data distribution. The generative module can be seen as a team of counterfeiters trying to produce fake currency. The discriminative module can be seen as the police trying to detect the fake currency. Competition in this game drives both teams to improve their methods until the fake currency is distinguishable from the genuine articles. The purpose of GAN is to narrow the gap between the model distribution and data distribution through sample-level adversarial training.

More importantly, compared to the previous loss in the pixel level or feature level, a GAN creates a domain-level loss, which provides a better solution to many problems. At present, GANs have been well applied in the fields of image generation, image super-resolution, text-to-image generation, image-to-image translation, and so on.

### 2.3. Unsupervised Image-to-Image Translation

Since GANs propose a novel loss from a domain perspective, unsupervised image-to-image translation work has achieved great success. CycleGAN [15], discoGAN [16], and dualGAN [17] separately applied an image converter for each domain to learn image translation through the proposed cycle consistency constraints. In order to further strengthen the constraints, DistanceGAN [18] proposed distance loss, forcing the distance between two pictures in domain A to be equal to the distance between the two translated pictures in domain B. Cycada [19] combined feature-level loss and pixel-level loss to achieve the alignment of the source domain image and the target domain image. TraVeLGAN [20] proposed a Siamese network that can be used to replace the cycle consistency loss, thereby reducing the complexity and training cost of the model. The Siamese network is used to learn the advanced semantic features of the image to ensure that the translated image is similar to the original domain image. In addition, PatchGAN [10] applied a Markovian discriminator to make the generator pay more attention to the details of the generated samples. What the Markovian discriminator does is cutting the image into patches and classifying whether each patch is real or fake.

In our design, the generating module takes in spliced samples and outputs realistic word samples, just like the unsupervised image-to-image translation. To some extent, our work can benefit a lot from the above literature.

### 2.4. Scene Text Recognition

In our work, we need to design an air-writing recognition network to demonstrate the effectiveness of the proposed method. The recognition task of air-written words is similar to the scene text recognition. In traditional text recognition methods [21,22], the task is generally divided into three steps: preprocessing, character segmentation, and character recognition. Character segmentation is considered to be the most challenging part due to the complex background and so on. It also greatly limits the performance of the whole recognition system.

In the era of deep learning, in order to avoid character segmentation, two main techniques are adopted, namely the Connectionist Temporal Classification (CTC) [23] and the Attention mechanism [24,25]. The first application of the CTC in text recognition can be traced to the handwriting recognition system of Graves et al. [26]. Now, this technique is widely adopted in scene text recognition [27,28,29].

In our work, we design a CRNN (Convolutional Recurrent Neural Network) [28] combined with CTC to verify the superiority of our method. Details about the network structure will be introduced in Section 4.1.

## 3. Methodology

We propose a novel two-stage synthesis method for in-air handwritten words. The proposed method can synthesize realistic samples under the condition of limited in-air handwritten character samples and word samples. The synthesis method proposed contains two modules. (1) Splicing module: The splicing module splices the air-written characters according to the order of characters in the specified word that comes from the corpus. The splicing module is not trainable. It consists of a couple of steps, which will be introduced in Section 3.2. (2) Generating module: The generating module takes the output of the splicing module as input and outputs the generated samples. A well-trained generating module generates realistic word samples, which could be used to train a recognition network. The details of the generating module will be introduced in Section 3.3.

For the convenience of the following description, we refer to the output samples of the splicing module as spliced samples and the output samples of the generating module as generated samples. The domain of spliced samples is called the spliced domain, and the domain of the real word samples is called the real word domain. The generating module mentioned above is actually a generator in a GAN. It takes in spliced samples and outputs realistic word samples. In other words, it learns the mapping from the spliced domain to the real word domain through adversarial training.

### 3.1. Motivations

The task of recognizing air-written words is similar to scene text recognition. In scene text recognition, a recognition network with good generalization performance not only learns to recognize a single character, but also learns a combination of characters (also called corpus information). Therefore, in the air-writing word synthesizer, we introduce a word set as a corpus to guide the splicing of character samples. The corpus can ensure that the synthetic dataset contains a variety of character combinations. Since the spliced samples are not realistic enough at the junctions, it is necessary to use adversarial training to modify the junctions of the spliced samples.

What the generating module does is somewhat similar to unsupervised image-to-image translation. However, when dealing with time series data, there are still several challenges to be solved.

Lack of paired training data: Usually, paired samples can provide a good guide for the model. However, in this synthesis task, a pairwise database requires the collector to record the start and end points of each character in the word while writing the words in the air. This is nearly impossible in practice. To solve this problem, cycle consistency is adopted.

Handling time series data. The in-air handwritten word samples based on inertial sensors are six-dimensional time series data. Due to the difference in the writing speed and the number of characters of different words, the duration of different word samples varies greatly. Interpolation or padding is usually used to normalize the length of time series data. However, when the duration varies greatly, interpolation or padding is not the best choice. In our work, we adopt U-Net as the backbone of the generator and a Markovian discriminator [10] as the discriminator of our proposed network to solve the above problem.

### 3.2. Splicing Module

**Figure 3:**
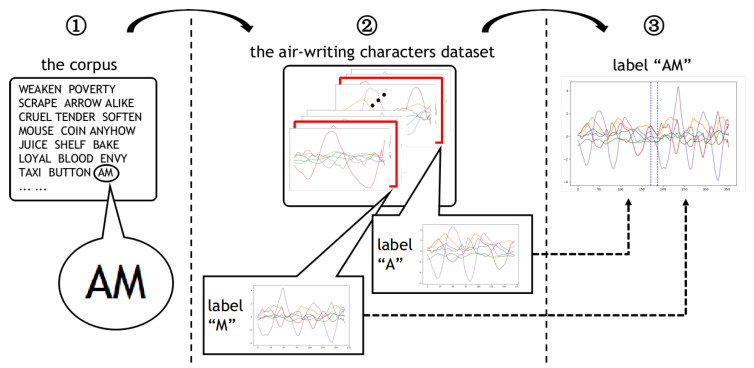
Pipeline of the splicing module. 1. The word “AM” is selected from the corpus. 2. A character sample labeled “A” and a character sample labeled “M” are picked from the air-writing character dataset. 3. The two character samples mentioned above are stitched together, but a gap is left between them. Then, the gap is filled using linear interpolation.

The splicing module produces spliced samples through the process shown in Figure 3. In the splicing process, an air-writing character dataset and a word set (also called corpus) are needed. A word set is a collection of words. It can be a “.txt” file with many words in it. Through the splicing module, we can get a less real spliced sample.

The pipeline of the splicing module can be divided into three steps, as shown in Figure 3. For example, first, we randomly select the word “AM” from the corpus. Then, since “AM” is composed of “A” and “M”, an inertial sample labeled “A” and an inertial sample labeled “M” are randomly selected from the character dataset. Finally, we stitch the two character samples together but leave a gap between them, and then fill the gap using linear interpolation. In our experimental setting, we set the length of the gap to 20.

### 3.3. Generating Module

**Figure 4:**
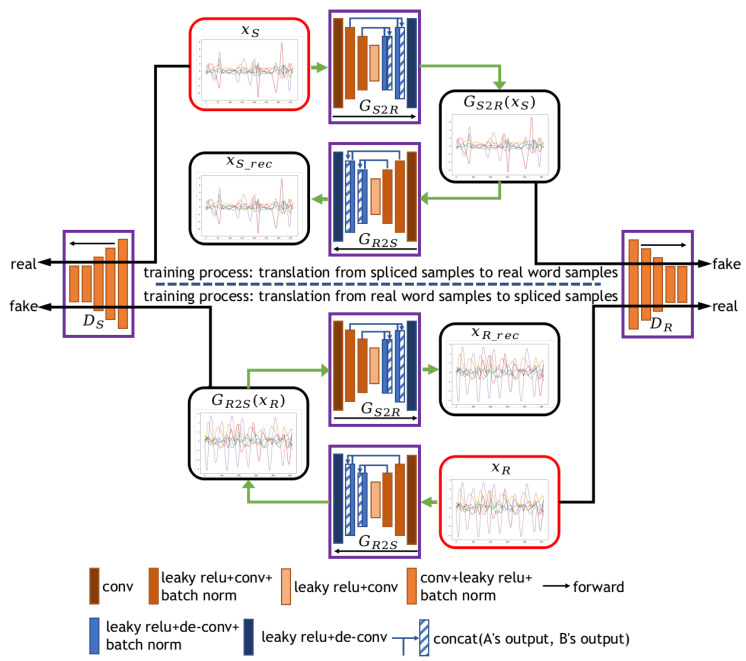
Training process of the generating module. The generating module refers to $G_{S2R}$. $x_{S}$ and $x_{R}$ in the red boxes denote input samples from the spliced domain and the real word domain. $G_{S2R}$ denotes the generator that maps from *S* domain to *R* domain. $G_{R2S}$ denotes the generator mapping from *R* domain to *S* domain. $D_{S}$ and $D_{R}$ denote discriminators in each domain.

The generating module is actually a generator of the GAN. In order to better train the generating module, we specially designed an adversarial network, as shown in Figure 4. Note that the generating module in Figure 1 refers to generator $G_{S2R}$.

As shown in Figure 4, the network we designed consists of two generators ($G_{S2R}$ and $G_{R2S}$) and two discriminators ($D_{S}$ and $D_{R}$). Let *S* and *R* denote the spliced domain and the real word domain, respectively. $G_{S2R}$ is used to learn the mapping from *S* domain to *R* domain, and $G_{R2S}$ is used to learn the mapping from *R* domain to *S* domain. $D_{S}$ and $D_{R}$ represent the discriminators of *S* domain and *R* domain, respectively. They are used to distinguish whether the input sample is real or fake.

For the conversion from *S* domain to *R* domain, given a labeled spliced sample $x_{S}$, $G_{S2R}$ maps $x_{S}$ into $G_{S2R}\left(x_{S}\right)$, which means the sample is translated into *R* domain. $G_{S2R}\left(x_{S}\right)$ is shared by two parts. Firstly, $G_{R2S}$ uses it to produce a reconstructed sample $x_{S_{rec}}=G_{R2S}\left(G_{S2R}\left(x_{S}\right)\right)$. Secondly, the discriminator $D_{R}$ uses it to distinguish whether the sample is real. The conversion from *R* domain to *S* domain is similar to the above conversion.

#### 3.3.1. Architecture Details

We assign a generator and a discriminator to each domain. Figure 4 illustrates the architecture of generators and discriminators. In our design, the two generators $G_{S2R}$ and $G_{R2S}$ adopt the U-Net as the backbone. Each generator consists of a contracting path for capturing context and a symmetric expanding path for generating samples. Among them, the contracting path contains eight convolutional layers and the expanding path contains eight de-convolutional layers. As shown in Figure 4, the input of each de-convolutional layer is a feature map that concatenates the output of the previous layer with the feature map of the same size in the contracting path. Both generators are fully convolutional networks, which are very suitable for handling variable-length input samples.

In Figure 4, the two discriminators $D_{R}$ and $D_{R}$ are Markovian discriminators. Each discriminator consists of five convolutional layers. The first four convolutional layers are used to downsample the input samples. The last convolutional layer compresses feature maps with multiple channels into feature maps with one channel. The average value of the last feature map is output as the final score. There are two reasons for using the Markovian discriminator. Firstly, the length of the air-writing word samples varies greatly. In this case, padding or interpolation performs badly in the GAN. A fully convolutional network can solve this problem well. Secondly, since the splicing samples are not real enough at the junctions, we hope that the GAN model can pay more attention to the details of the generated samples. In the last feature map output by the Markovian discriminator, each point corresponds to a patch in the input sample. The Markovian discriminator tries to classify whether each patch is real or fake. In other words, the Markovian discriminator pays more attention to the details of the generated samples.

In addition, we have modified the structure of these models to handle time series input. We also adopt a strip-shaped convolutional kernel in all convolutional and de-convolutional layers. The kernel size of each layer in the generators and discriminators is 9×3.

In the last feature map output by the discriminator, each point corresponds to a patch of length 33 in the input sample. Therefore, in the splicing module, the gap between the two samples should not exceed the size of the discriminator’s receptive field. In the experimental setting, we set the length of the gap to 20.

#### 3.3.2. Loss Functions

In this section, we will only introduce the loss functions in the conversion from *S* domain to *R* domain. The conversion from *R* domain to *S* domain uses similar loss functions. Since the training of the generating module is based on the GAN, we introduce the loss functions of the discriminator and the generator.

The loss function of the discriminator is as follows.

(1) $$\begin{array}{c} L_{disc}=L_{adv/disc}, \end{array}$$

where $L_{adv/disc}$ is the adversarial loss of the discriminator.

The loss function of the generator is mainly composed of three parts. In our attempt, λ=10 and β=1 perform best.

(2) $$\begin{array}{c} L_{gen}=L_{adv/gen}+\lambda *L_{cyc}+\beta *L_{dist}, \end{array}$$

where $L_{adv/gen}$ is the adversarial loss of generator, $L_{cyc}$ is the cycle loss, and $L_{dist}$ is the distance loss.

(1) Adversarial loss: The $G_{S2R}$ (generating module) learns a mapping from *S* domain to *R* domain by adversarial training. Unlike the original GAN, this proposed model uses a fully convolutional Markovian discriminator. As mentioned above, the Markovian discriminator tries to classify if each patch is real or fake across input samples and averages all responses to provide its final output.

The adversarial loss we used is as follows:

(3) $$\begin{array}{cc} L_{adv/disc}= & E_{x_{R}∼p_{R}}\left[{\left(D_{R}\left(x_{R}\right)-1\right)}^{2}\right]+E_{x_{S}∼p_{S}}\left[D_{R}{\left(G_{S2R}\left(x_{S}\right)\right)}^{2}\right] \end{array}$$

(4) $$\begin{array}{cc} L_{adv/gen}= & E_{x_{S}∼p_{S}}\left[{\left(D_{R}\left(G_{S2R}\left(x_{S}\right)\right)-1\right)}^{2}\right], \end{array}$$

where $x_{R}$ denotes real air-writing word samples, $x_{S}$ denotes spliced air-writing word samples, $D_{R}$ is the discriminator in the real word domain, and $G_{S2R}$ is the generator that inputs spliced samples and outputs generated samples.

(2) Cycle consistency loss: The adversarial loss ensures that samples with the distribution of *S* are translated to samples with the distribution of *R*. However, there are many possible mappings. We apply cycle consistency loss to force the mapping from *S* to *R* to be the inverse process of the conversion from *R* to *S*, which reduces the number of admissible mappings. To a great extent, cycle consistency loss ensures stable training.

The following is the cycle consistency loss:

(5) $$\begin{array}{cc} L_{cyc}= & E_{x_{S}∼p_{S}}\left[{\left(G_{R2S}\left(G_{S2R}\left(x_{S}\right)\right)-x_{S}\right)}^{2}\right], \end{array}$$

where $x_{S}$ denotes spliced air-writing word samples, $G_{S2R}$ is the generator that inputs spliced samples and outputs generated samples, and $G_{R2S}$ is the generator that maps samples from *R* domain to *S* domain.

(3) Distance loss: To further maintain the semantic consistency between *S* domain and *R* domain, distance loss is adopted. The distance loss forces the distance between two samples in *S* domain to be preserved in the mapping to *R* domain.

The distance loss is as follows:

(6) $$\begin{array}{cc} L_{dist}= & E_{x_{i},x_{j}∼p_{S}}\left[|\right|x_{i}-x_{j}{\left|\right|}_{1}-\left|\right|G_{S2R}\left(x_{i}\right)-G_{S2R}\left(x_{j}\right){\left|\right|}_{1}], \end{array}$$

where $x_{i}$ and $x_{j}$ are spliced air-writing word samples, and $G_{S2R}$ is the generator that inputs spliced samples and outputs generated samples.

#### 3.3.3. Training Strategy

The air-writing word synthesizer is a two-stage synthesis strategy. Specifically, it includes a splicing module and a generating module. The splicing module is not trainable, while the generating module is end-to-end trainable. In this section, we introduce the specific training strategy of the generating module in detail.

Since the generating module is a part of the GAN, we adopt an alternate training strategy. We alternate between *k* steps of optimizing the two discriminators and one step of optimizing the two generators. When training $D_{S}$ and $D_{R}$, the parameters of $G_{R2S}$ and $G_{S2R}$ remain fixed. When training $G_{R2S}$ and $G_{S2R}$, the parameters of $D_{S}$ and $D_{R}$ remain fixed. Note that we set k=1 to keep the balance of adversarial training. With the ADAM [30] optimization method, we set the learning rate to 2e-4 and batch size to 64. Finally, our model is trained on NVIDIA-1080Ti GPU, so it is GPU-accelerated.

## 4. Experiment

In this section, we aim to prove that the air-writing synthesizer can greatly improve the performance of the word recognition model. Therefore, we designed a word-IAHR model and conducted experiments on a public data set named 6DMG [31].

### 4.1. Word-IAHR Model

**Figure 5:**
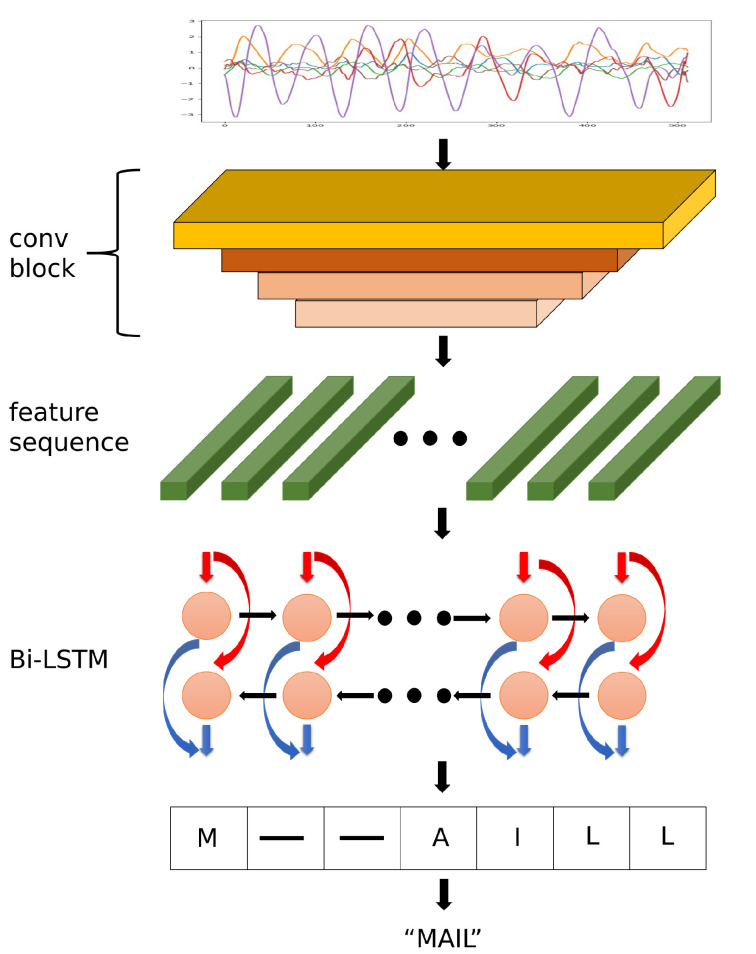
Architecture of the word-level in-air handwriting recognition (word-IAHR) model.

The word-IAHR model contains five convolutional layers for downsampling and two layers of bidirectional LSTM (Long-Short Term Memory) [32] for capturing long-distance information. Like the generating module in the air-writing word synthesizer, in order to better process the time series samples, we adopt a strip-shaped convolutional kernel in the convolutional layers. The kernel size of each layer is 9×3. Finally, the model is trained by CTC loss. We use an ADAM optimizer to update the network with a learning rate of $1\times 10^{-4}$ and a batch size of 256. Figure 5 illustrates the architecture of the word-IAHR model we used for experiments.

### 4.2. Dataset

The 6DMG dataset contains two subsets, namely the air-writing character dataset (char-6DMG) and the air-writing word dataset (word-6DMG). Both the character dataset and the word dataset are collected within a fixed range. A total of 25 volunteers participated in the collection of the dataset. Ten volunteers participated in the collection of both the char-6DMG dataset and the word-6DMG dataset, while the remaining 15 writers only participated in the char-6DMG dataset collection.

**Figure 6:**
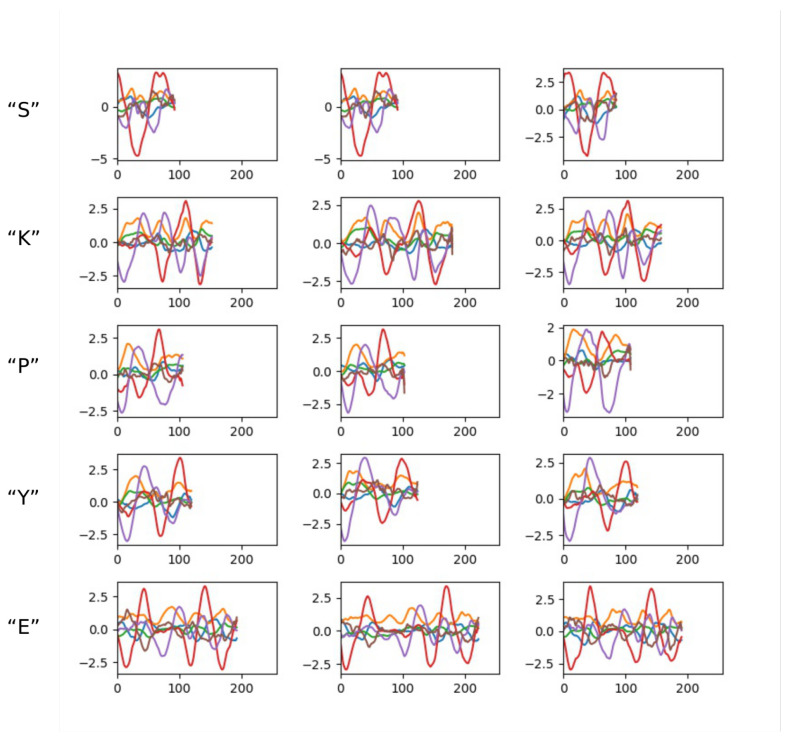
Samples in the air-writing character dataset (char-6DMG) dataset. Samples in each row belong to the same category.

The char-6DMG is composed of 26 uppercase letter categories. It contains 6500 samples in total, with an average of 250 samples in each category. The number of samples in each category is approximately equal. In the char-6DMG dataset, most samples’ lengths vary between 100 and 200. The maximum length of a character sample is 297, and the minimum is 89. Figure 6 shows some examples from the char-6DMG dataset. Samples of the same category or samples of different categories have different lengths.

**Figure 7:**
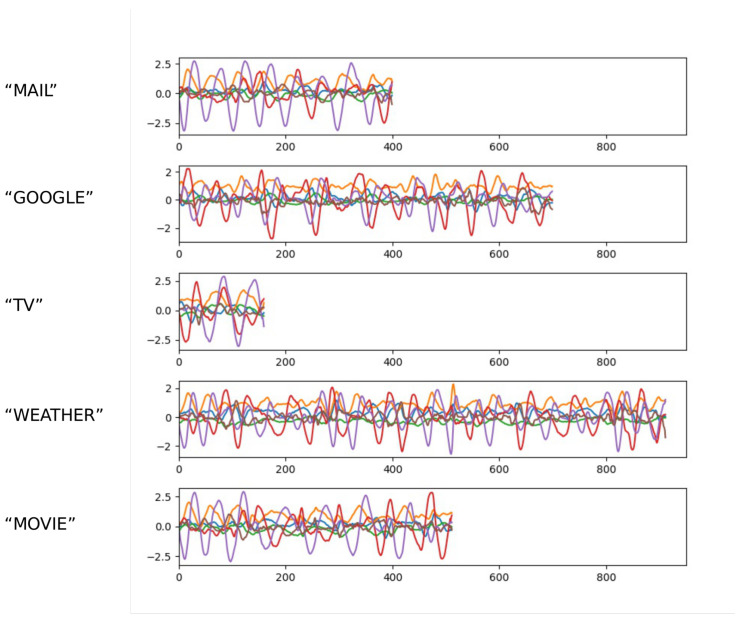
Samples in the air-writing word dataset (word-6DMG) dataset.

The word-6DMG dataset contains 1230 samples in total, including 40 categories of words. The number of samples in each category is approximately equal. Figure 7 shows some examples in the word-6DMG dataset. As shown in Figure 7, the lengths of air-writing words vary greatly.

In the word-6DMG dataset, most samples’ lengths vary between 300 and 700. The number of characters in most word samples is usually between three and seven, but the longest word sample contains nine characters and is 1512 in length. The shortest word sample contains only two characters and is 145 in length.

Word set: Due to the particularity of our method, we use a word set (corpus). The word set is a text file consisting of 2000 common words. In our proposed method, the words in the word set are used as labels for synthetic samples. It is worth noting that the corpus we used in the training process does not contain the 40 words in the word-6DMG dataset.

Synthetic dataset: According to the method proposed in this work, if we suppose we want to synthesize a sample labeled “CAT”, we need to select the corresponding character samples from the char-6MDG dataset. Since each category in the char-6DMG dataset contains 250 samples, theoretically, in the synthetic dataset, there are a total of $250^{3}$ different samples in the “CAT” category. It is a very large dataset, which will bring challenges in terms of data storage and data reading.

Therefore, we use an online synthesis strategy instead of synthesizing the dataset in advance. The pipeline of the air-writing word synthesizer can be seen in Figure 1. In the following part, the synthetic dataset refers to data synthesized by the proposed online synthesis method.

### 4.3. Dataset Pre-Processing

We apply a moving-average filter with a window length of 3 to filter out the noise in both the char-6DMG dataset and the word-6DMG dataset. For samples whose length exceeds 700 in the word-6DMG dataset and synthetic dataset, we resize their length to 700.

### 4.4. Experiments and Results

In order to demonstrate the superiority of our method, we conduct the following experiments under two principles. One is user-independent, which means that users in the test data will never appear in the training data. The other is user-mixed, which means that when the dataset is divided into folds, each fold is composed of samples recorded by each user.

#### 4.4.1. Experiments on the Word-6DMG Dataset

In scene text recognition, a well-performed recognition network usually requires millions of samples for training. A recognition model can learn more corpus information from a larger dataset. Therefore, we design the following four experiments to verify whether a large dataset is necessary in the word-IAHR task.

- Non-cross-label experiments: As shown in the first two rows of Table 1, we take 80% of the word samples in 6DMG as the training set and the rest as the test set. When dividing the dataset, it is necessary to ensure that samples of the same categories can only appear in the same set and the labels of the training set cover all 26 capital letters. So, we call these non-cross-label experiments. For example, all samples labeled “AM” can only appear in either the training set or test set. That is, we take 32 categories out of 40 as the training set and the remaining eight categories as the test set. The training set contains 984 samples, and the test set contains 246 samples.
- Cross-label experiments: The last two rows of Table 1 show the settings of the cross-label experiments. We take 80% of the word samples in 6DMG as the training set and the rest as the test set. When dividing the dataset, we make sure both the training set and test set contain all 40 categories.

We report the results of these four experiments in Table 2. Whether it is user-independent or user-mixed, the accuracy of the non-cross-label experiment is close to 0.0%. For cross-label experiments, under user-mixed principle, the word-level accuracy is 98.8%. Under the user-independent principle, the word-level accuracy is 88.1%.

Based on the experimental results, we can draw the following conclusions.

- A word-IAHR model trained on a small dataset performs badly. It is almost impossible to identify samples that do not appear in the training set even if the labels of the training set have covered all 26 capital letters. A word-IAHR model with good generalization performance requires a large amount of data for training.
- Even trained on such a small dataset, the model can still recognize samples whose labels have already appeared in the training set, regardless of whether it follows the principle of being user-mixed or user-independent. This is because samples of the same word category have similar features.

#### 4.4.2. Quality Testing

According to the results in Table 2, we can know that a recognition model can easily recognize a sample whose label is the same as that of the training sample.

In this section, we want to verify the quality of the synthetic dataset. Based on the experimental results of Table 2, we can infer that synthetic samples can be considered to be of high quality if they are correctly recognized by the word-IAHR model.

In terms of the synthetic dataset used in this section, we use the labels of the word-6DMG dataset (40 words) as a corpus to synthesize a dataset. We refer to this synthetic dataset as the “synthetic dataset of 40 words”. It includes 40 categories and 1230 samples.

As shown in Table 3, we conducted quality testing based on the user-mixed and user-independent principles. For comparison, the well-trained models (rows 3 and 4 of Table 2) are adopted. In the quality testing based on the user-mixed principle, the training set is the same as the training set used in the third row of Table 1. In the quality testing based on the user-independent principle, the training set is the same as the training set used in the fourth row of Table 1. We take the “synthetic dataset of 40 words” as the test set. It is of great significance that the “synthetic dataset of 40 words” shares a label space with the word-6DMG dataset.

We report the results in Table 4. In the quality testing based on the user-mixed principle, the word-level recognition accuracy is 86.7%. In the quality testing based on the user-independent principle, the word-level recognition accuracy is 72.3%. Compared with the results in the last two rows of Table 2, the accuracy is slightly lower, but this also shows that the synthetic samples are very similar to the real word samples. The results prove that synthetic samples are of high quality.

#### 4.4.3. Verification of Generalization Performance

As shown in Table 5, we design generalization performance experiments based on the user-mixed and user-independent principles to verify the effectiveness of the proposed method. We use the synthetic dataset as the training set and the word-6DMG dataset as the test set. It is of great significance that not every category in the training set can appear in the test set.

We report the results in Table 6. In the experiment under the user-mixed principle, the word-level recognition accuracy is 62.3%. In the experiment under the user-independent principle, the recognition accuracy is 44.6%. Compared with the non-cross-label results (the first two rows in Table 2), the deep model trained on the synthetic dataset improves performance by 62% and 44%, respectively. With the help of the air-writing word synthesizer, the recognition model can achieve an excellent performance improvement in samples whose labels are fresh to the model. These experimental results demonstrate the success of our method.

The air-writing word synthesizer can synthesize a large number of samples under the condition of limited character samples and word samples. In our design, we have greatly enriched the combination of characters by introducing a corpus. This makes the recognition model not only learn to recognize a single character, but also learn combinations of characters (also known as the corpus information of the dataset). Generally speaking, a word-IAHR model can benefit a lot from the corpus information of the dataset.

### 4.5. Discussion on the Splicing Module and Generating Module

The air-writing word synthesizer is a two-stage synthesis method. In this section, we intend to show the respective effects of the splicing module and the generating module.

**Figure 8:**
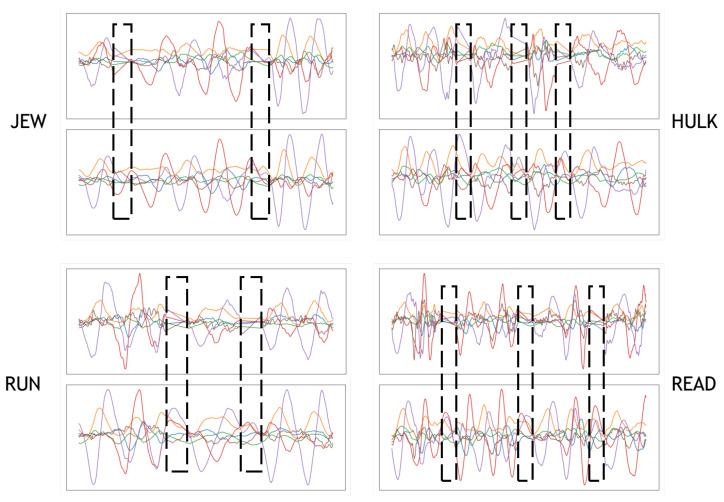
Outputs of the splicing module and generating module. There are two sample outputs for each word: The upper one is the wave of the spliced sample, and the lower one is the wave of the synthesized sample.

In Figure 8, we illustrate several word sample outputs of the splicing module and the generating module (i.e., “JEW”, “RUN”, “HULK”, and “READ”). For example, we take the word “JEW” as the target word. First, we pick character samples “J”, “E”, and “W” from the char-6DMG. Then, we splice them using the splicing module to obtain the spliced sample. Finally, the generating module takes in the spliced sample labeled “JEW” and outputs the synthetic sample.

In Figure 8, the dashed box represents the junctions between the characters in the spliced word sample. The characters in the spliced samples are connected by linear interpolation. We can see that, after processing by the generating module, the junctions become more natural. We believe that adversarial training helps a lot.

The main disadvantage of our model is the dependence on datasets. Our method requires that the character dataset and word dataset used for training must be collected under the same rules. For example, the users must write within a fixed range while collecting data. If the two datasets are collected under different rules, the air-writing word synthesizer will not perform so well. In addition, using linear interpolation to splice characters may not be the optimal solution. In the future, we will try to model the strokes between characters mathematically. In this way, we can figure out a better solution for splicing characters. In addition, we will try to apply the method to various datasets. For example, we can improve our method so that it can be used even if the data collection rules are different.

### 4.6. Evaluation of Computational Efficiency

In this section, we evaluate the computational efficiency of our proposed method. Note that the air-writing word synthesizer is a synthesis method that works in the training phase. This synthesis method can greatly improve the generalization performance of the recognition model, but it will not slow down the inference process.

In Table 7, we report the computational efficiency of our proposed method. The “synthesis (per sample)” refers to the average time taken to synthesize one sample by the air-writing word synthesizer. The “recognition (per sample)” refers to the average time taken to recognize one sample. We argue that our approach is conducive to devices with limited computing resources and has a wide range of application scenarios.

## 5. Conclusions

In-air handwritten word recognition usually suffers from insufficient data. In this work, we propose a new two-stage synthesis method (named the air-writing word synthesizer) for in-air handwritten words to solve this problem. The proposed method includes two components: a splicing module and a generating module. In the splicing module, a corpus is introduced to provide guidance for word splicing. In the generating module, adversarial learning is introduced to guide word synthesis. In addition, the network architecture is carefully designed to handle time series.

Experiments on public datasets show that the proposed method can synthesize samples of high quality. Most air-writing recognition tasks can benefit from the air-writing word synthesizer.

The main disadvantage of our model is its dependence on datasets. Our method requires that the character dataset and the word dataset used for training are collected under the same rules. For example, the users must write within a fixed range while collecting. If the two datasets are collected under different rules, the air-writing word synthesizer will not perform so well. In addition, linear interpolation is adopted to splice characters, which may not be the optimal solution.

In the future, we will try to mathematically model the strokes between characters in a word. In this way, we may figure out a better plan for splicing characters. In addition, we will try to apply the method to multiple datasets. For example, we can improve our method so that it can be used even if the data collection rules are different.

## Figures and Tables

**Table 1:** Experimental settings for the word-6DMG dataset.

Experiments	Number of Categories in Training Set	Number of Categories in Test Set	Number of Categories in Both Training Set and Test Set
Non-cross-label (user-mixed)	32	8	0
Non-cross-label (user-independent)	32	8	0
Cross-label (user-mixed)	40	40	40
Cross-label (user-independent)	40	40	40

**Table 2:** Experimental results for the word-6DMG dataset.

Experiments	Word-Level Accuracy
Non-cross-label (user-mixed)	0.02
Non-cross-label (user-independent)	0.00
Cross-label (user-mixed)	0.988
Cross-label (user-independent)	0.881

**Table 3:** Experiment setup for quality testing.

Experiments	Training Set	Number of Categories in Trainig Set	Test Set	Number of Categories in Test Set	Number of Categories in Both Training Set and Test Set
Quality testing (user-mixed)	word-6DMG	40	“synthetic dataset of 40 words”	40	40
Quality testing (user-independent)	word-6DMG	40	“synthetic dataset of 40 words”	40	40

**Table 4:** Experimental results of quality testing.

Experiments	Word-Level Accuracy
Quality testing (user-mixed)	0.867
Quality testing (user-independent)	0.723

**Table 5:** Experimental settings for generalization performance experiments.

Experiments	Training Set	Number of Categories in Trainig Set	Test Set	Number of Categories in Test Set	Number of Categories in Both Training Set and Test Set
Generalization performance experiment (user-mixed)	synthetic dataset	2000	word-6DMG	40	0
Generalization performance experiment (user-independent)	synthetic dataset	2000	word-6DMG	40	0

**Table 6:** Experimental results of generalization performance experiments.

Experiments	Word-Level Accuracy
Generalization performance experiment (user-mixed)	0.623
Generalization performance experiment (user-independent)	0.446

**Table 7:** Computational efficiency of our proposed method.

Item	Average Time (ms)
Synthesis (per sample)	1.96
Recognition (per sample)	19.09
